# Serum Concentrations of KL-6 in Patients with IPF and Lung Cancer and Serial Measurements of KL-6 in IPF Patients Treated with Antifibrotic Therapy

**DOI:** 10.3390/cancers13040689

**Published:** 2021-02-09

**Authors:** Miriana d’Alessandro, Laura Bergantini, Paolo Cameli, Maria Pieroni, Rosa Metella Refini, Piersante Sestini, Elena Bargagli

**Affiliations:** Respiratory Diseases and Lung Transplantation Unit, Department of Medical and Surgical Sciences & Neurosciences, Siena University Hospital, 53100 Siena, Italy; bergantini@student.unisi.it (L.B.); cameli3@student.unisi.it (P.C.); pieronim@unisi.it (M.P.); refini@unisi.it (R.M.R.); sestini@unisi.it (P.S.); bargagli2@unisi.it (E.B.)

**Keywords:** idiopathic pulmonary fibrosis, antifibrotic therapies, Krebs von den Lungen-6, lung cancer, pulmonary fibrosis associated with autoimmune diseases

## Abstract

**Simple Summary:**

Evaluation of the prognostic significance of serial measurements of serum concentrations of Krebs von den Lungen-6 (KL-6) showed that an annual increment in KL-6 exceeding a threshold amount was an independent risk factor for progressive disease and poor prognosis. No literature data are available on long-term KL-6 measurements in the follow-up of IPF patients treated with Nintedanib. The aim of this study is to serially analyze KL-6 in idiopathic pulmonary fibrosis (IPF) patients after 24 months of Nintedanib and to investigate its biomarker potential in patients with IPF and lung cancer with respect to fibrotic hypersensitivity pneumonitis patients, pulmonary fibrosis associated with autoimmune diseases group and healthy controls.

**Abstract:**

Background: Krebs von den Lungen-6 (KL-6) was suggested as ILD biomarker including idiopathic pulmonary fibrosis (IPF). Lung cancer is one of the most severe comorbidity of IPF patients. This study aims to serially analyze KL-6 in IPF patients after 24 months of Nintedanib and to first investigate the biomarker behavior in IPF associated with adenocarcinoma. Materials and methods: One hundred and forty-two ILD patients (median (IQR), 69 (63–75) years; 86 males) were retrospectively enrolled. Serial serum samples were collected from IPF patients before starting antifibrotic therapy and after 12 months. Serum KL-6 levels were measured by KL-6 reagent assay (Fujirebio Europe, UK). Results: Increased KL-6 concentrations were identified in IPF-LC patients than IPF, fibrotic hypersensitivity pneumonitis, and pulmonary fibrosis associated with autoimmune disease groups. A cut-off value was calculated to distinguish IPF and IPF-LC patients. IPF patients monitored for 24 months with Nintedanib showed persisted increased levels of KL-6 with a progressive decline of FVC percentages. Conclusion: This preliminary study offers a first demonstration that very high serum concentrations of KL-6 in IPF-LC patients are associated with poor prognosis. Moreover, serial evaluation of serum KL-6 in IPF patients over 24 months of Nintedanib treatment revealed that most patients experienced a stabilization of lung function parameters and of serum concentrations of KL-6.

## 1. Introduction

Krebs von den Lungen-6 (KL-6) is a high-molecular-weight (200 kDa) glycoprotein predominantly expressed in the lungs by injured and regenerating alveolar type II cells [1,2]. KL-6 was first suggested as a serum biomarker for lung, breast, and pancreatic cancer, but showed lower diagnostic accuracy than other tumor markers, such as carcinoembryonic antigen (CEA), a reliable predictor of treatment response in non-small-cell lung cancer [3]. Subsequently, KL-6 was proposed as a diagnostic biomarker for differentiating interstitial lung diseases (ILDs) and predicting response to antifibrotic therapy [4,5,6]. A cut-off value of 465 U/mL was recently established to distinguish ILD patients from the healthy subjects and patients with other non-fibrotic lung diseases [7], however the potential value of KL-6 as a biomarker in the differential diagnosis of ILDs is still limited [8,9]. In 1999, the Japanese Health Insurance Program approved serum KL-6, measured by a chemiluminescent enzyme immunoassay (CLEIA), as a diagnostic marker of ILDs. The assay is not yet available in Western European countries, including Italy.

Serum concentrations of KL-6 have been found elevated in ILDs, including idiopathic pulmonary fibrosis (IPF), a disease characterized by alveolar epithelial cell damage and progressive interstitial thickening [5,8,9,10]. IPF is a chronic progressive ILD of unknown etiology, occurring primarily in older adults. It is limited to the lungs and characterized by a radiological and histopathological pattern of usual interstitial pneumonia [11,12]. IPF shares various genetic, molecular, and cell processes with lung cancer [13]. Today, an “antifibrotic” approach is widely accepted and the only drugs approved for the treatment of IPF are pirfenidone and Nintedanib [14,15]. Nintedanib, a tyrosine-kinase inhibitor, was first developed as an anticancer drug because it suppresses angiogenesis, but it was later recognized as an anti-fibrotic agent and approved for the treatment of IPF [16]. No single reliable biomarker is available to predict disease progression or response to treatment, or for differential diagnosis of IPF with respect to other fibrotic diseases, such as non-specific interstitial pneumonia and hypersensitivity pneumonitis [17,18]. Some authors have suggested that evaluation of serum concentrations of KL-6 at disease onset (above or below the cut-off 1000 U/mL) can predict survival in patients with IPF [8,19,20]. Fluctuations in KL-6 during follow-up of IPF patients have also been reported to have potential for predicting functional progression of the disease. Evaluation of the prognostic significance of serial measurements of serum concentrations of KL-6 showed that an annual increment in KL-6 exceeding a threshold amount was an independent risk factor for progressive disease and poor prognosis [4,5]. This finding sustains the fundamental value of serial monitoring of KL-6 during follow-up and of monitoring response to treatment.

To our knowledge, no data are available in the literature on long-term KL-6 measurements in the follow-up of IPF patients treated with Nintedanib. The aim of this study was to serially analyze KL-6 in IPF patients after 24 months of Nintedanib and to investigate its biomarker potential in patients with IPF and lung cancer with respect to fibrotic hypersensitivity pneumonitis, pulmonary fibrosis associated with autoimmune diseases patients and healthy controls.

## 2. Results

The clinical, demographic and immunological data is reported in Table 1. No significant differences in age, gender, or smoking history were observed between disease groups. Survival analysis showed that no IPF-LC patient survived more than 6 (IQR 4–8) months after serum collection.

**Figure 1:**
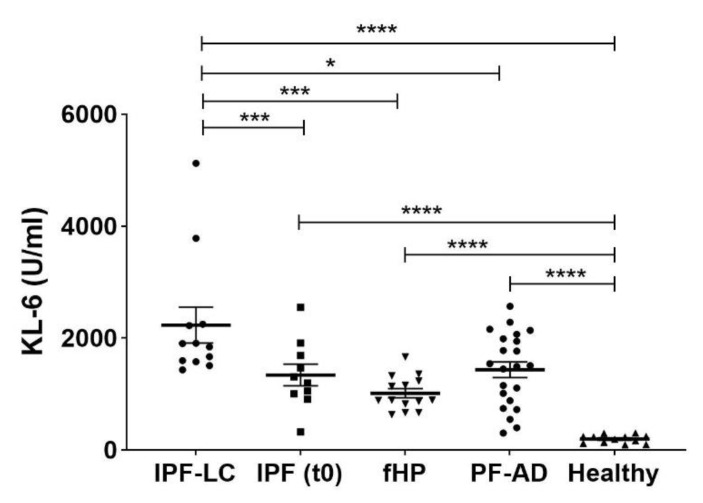
Serum KL-6 concentrations in the study population. Abbreviations: IPF, idiopathic pulmonary fibrosis; IPF-LC, idiopathic pulmonary fibrosis associated with lung cancer; fHP, fibrotic hypersensitivity pneumonitis; PF-AD, pulmonary fibrosis associated with autoimmune diseases; HC, healthy controls; KL-6, Krebs von den Lungen-6. * *p* < 0.05; *** *p* < 0.02; **** *p* < 0.0001.

**Figure 2:**
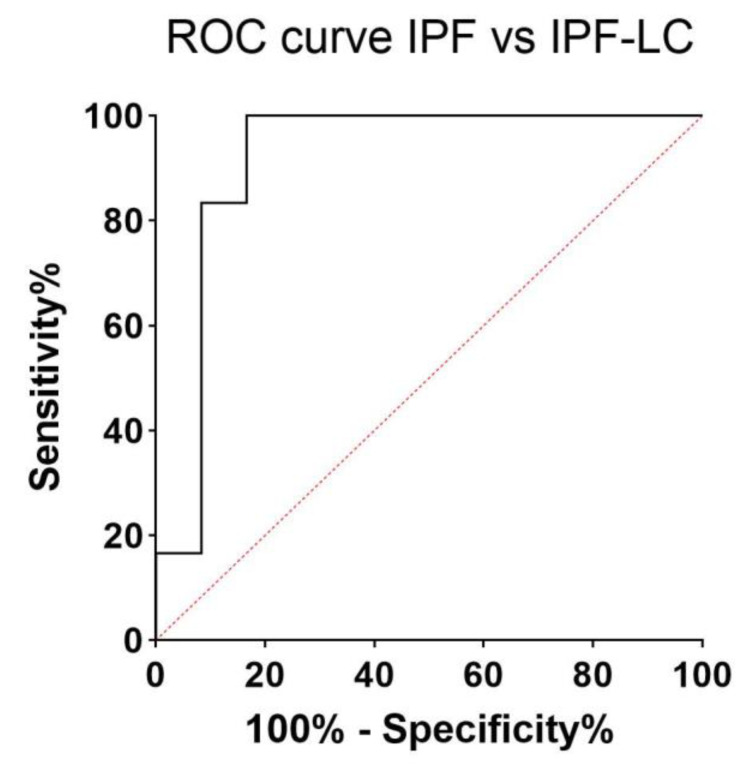
Receiver operating characteristic (ROC) curve analysis distinguished IPF and IPF-LC patients (AUC 92%; 95% CI 78–100; *p* = 0.0005), indicating a best cut-off value of 1370 U/mL (83% sensitivity, 92% specificity).

Serum concentrations of KL-6 were higher in patients than controls (Figure 1). The results of receiver operating characteristic (ROC) curve analysis to distinguish the healthy controls (HC) and disease subgroups are shown in Table 2. Serum concentrations of KL-6 were higher in IPF-LC than IPF patients at t0 (*p* = 0.0002) (Table 1) and ROC curve analysis distinguished these two groups (AUC 92%; 95% CI 78-100; *p* = 0.0005), indicating a best cut-off value of 1370 U/mL (83% sensitivity, 92% specificity) (Figure 2). Significant differences were observed between patients with IPF-LC and fibrotic hypersensitivity pneumonitis (fHP) (*p* = 0.0001) and healthy controls (*p* < 0.0001). ROC curve analysis of KL-6 levels distinguished IPF-LC and fHP patients (AUC 91.2%; 95%CI 79–100; *p* = 0.0003), indicating a best cut-off of 1395 U/mL (93% sensitivity, 92% specificity). Increased KL-6 concentrations were observed in IPF-LC patients than pulmonary fibrosis associated with autoimmune diseases (PF-AD) group (*p* = 0.0022) and ROC curve analysis distinguished these two groups (AUC 71%; 95% CI 54–88; *p* = 0.0436), indicating a best cut-off value of 1809 U/mL (68% sensitivity, 58% specificity). No differences in KL-6 concentrations emerged between IPF, fHP, and PF-AD patients.

**Figure 3:**
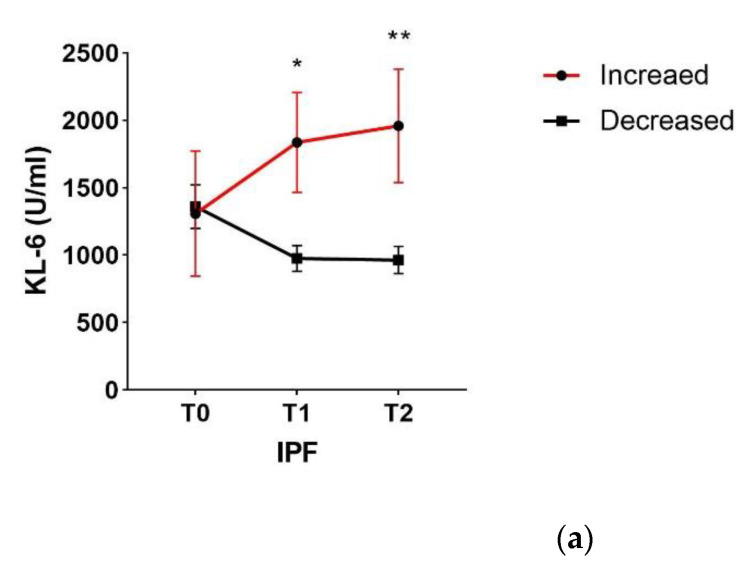

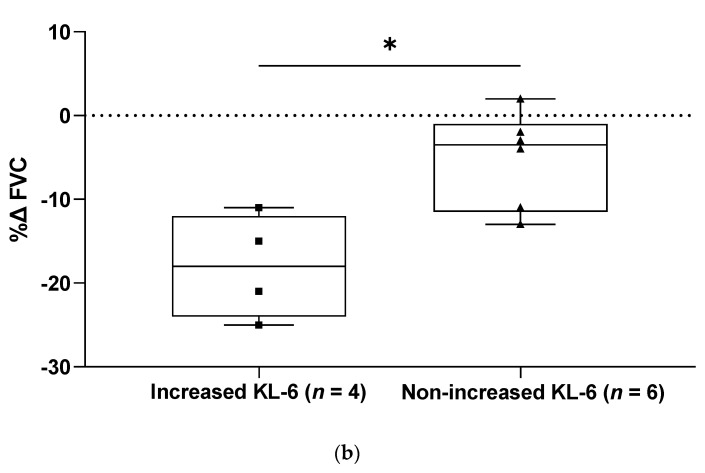
(**a**). After 12 and 24 months of Nintedanib treatment, serial concentrations identified 4/10 patients (40%) who showed a progressive increase in KL-6 levels during follow-up, and the other 6/10 (60%) who showed decreasing KL-6 concentrations. (**b**) Patients in the increased KL-6 group showed a significantly greater decline in %ΔFVC compared to those in the non-increased KL-6 group over the clinical course. * *p* < 0.05; ** *p* < 0.01. Abbreviations: IPF, idiopathic pulmonary fibrosis; KL-6, Krebs von den Lungen-6; FVC, forced vital capacity.

Serial concentrations of serum KL-6 in the IPF group treated with Nintedanib showed increased baseline KL-6 in 9/10 patients (90%). After 12 and 24 months of Nintedanib treatment, serial concentrations identified 4/10 patients (40%) who showed a progressive increase in KL-6 levels during follow-up, and the other 6/10 (60%) who showed decreasing KL-6 concentrations (Figure 3a).

A greater decrement in forced vital capacity (FVC) at 24-month follow-up was observed in patients who had persistently elevated KL-6 levels, whereas those with decreasing serial concentrations of KL-6 did not show a significant decrement in FVC. The relationship between baseline KL-6 concentrations and FVC percentages trend was showed in Figure 3b in which patients with increased KL-6 concentrations had a significantly greater decline in %ΔFVC compared to those with unincreased KL-6 concentrations during the clinical course (*p* = 0.0143).

## 3. Discussion

The present study aimed [1] to serially analyze KL-6 in IPF patients after 24 months of Nintedanib and [2] to investigate its biomarker potential in patients with IPF-LC with respect to fHP, PF-AD patients, and healthy controls.

KL-6 has been widely investigated as a specific marker of progressive fibrotic ILD, including IPF. Yokoyama et al. suggested that serum concentrations of KL-6 at disease onset (above or below a cut-off of 1000 U/mL) can predict survival of IPF patients [8]. Ishii et al. demonstrated in an IPF population that high baseline concentrations of KL-6 were related to significantly worse survival than low baseline concentrations [19]. Satoh et al. also associated serum KL-6 >1000 U/mL with poor prognosis of IPF patients [20], suggesting that elevated KL-6 levels may provide valuable information for phenotyping ILD patients at high risk of mortality [20]. Unfortunately, no data are available on the predictive value of KL-6 in IPF associated with lung cancer.

Fluctuations in serum concentrations of KL-6 during follow-up of IPF patients were recently suggested to have potential for predicting functional disease progression and response to treatment [4,5]. The prognostic significance of serial measurements of KL-6 was evaluated by Wakamatsu et al. in a pioneering study demonstrating that an annual increment in KL-6 exceeding a certain threshold was an independent risk factor for progressive disease and poor prognosis. These authors sustain the fundamental value of serial KL-6 monitoring in the follow-up of IPF [5]. A few months ago, our group performed serial measurements of serum KL-6 in IPF patients after 6 and 12 months of Nintedanib treatment, demonstrating that KL-6 may be a useful prognostic biomarker of IPF progression and response to Nintedanib. This biomarker was demonstrated to have clinical utility in monitoring IPF patients under specific antifibrotic treatments [4]. The study demonstrated that IPF patients treated with Nintedanib for one year maintained stable FVC percentages and KL-6 concentrations with respect to baseline [4]. The present study confirmed these results over a longer follow-up period: IPF patients were monitored for 24 months after starting Nintedanib. Interestingly, only those patients showing persistently elevated levels of KL-6 after two years of antifibrotic therapy showed a progressive decline in FVC percentages.

Lung cancer is a major comorbidity of IPF patients. To our knowledge, this is the first study to monitor serum concentrations of KL-6 in a cohort of IPF patients with adenocarcinoma. This preliminary population is of interest because higher KL-6 concentrations were recorded in IPF-LC patients than in the IPF and fHP groups, demonstrating that KL-6 may be a useful prognostic biomarker, predictive of IPF progression.

Very high KL-6 concentrations were first documented in the serum of IPF patients with histological evidence of lung cancer. The negative prognostic value of this result was clearly demonstrated by the fact that 2 years later, only 2/12 (16%) IPF-LC patients were still alive. Although the number of patients was limited, the demonstration of elevated KL-6 concentrations in the specific IPF-LC phenotype is of interest, because the prognosis of patients with this very rare association of lung diseases is unclear. Patients with IPF have five times the risk of the general population of developing lung cancer. Despite a few studies looking for relationships between these two severe lung diseases, diagnostic and therapeutic management of patients is still a challenge and no specific prognostic biomarker has yet been found.

## 4. Materials and Methods

### 4.1. Study Population

One hundred and forty-two ILD patients (median (IQR), 69 (63–75) years; 86 males), monitored at Siena Regional Referral Centre for Sarcoidosis and other Interstitial Lung Diseases, were retrospectively enrolled. We selected patients with IPF treated with Nintedanib for at least two years (*n* = 10; median (IQR), 77 (67–80) years; 8 males), IPF-LC with a histological diagnosis of adenocarcinoma (*n* = 12; median (IQR), 72 (65–75) years; 9 males), fHP with histological confirmation (*n* = 14; median (IQR), 67 (57–72) years; 8 males) and PF-AD (rheumatoid arthritis, *n* = 10, median (IQR), 65 (55–66) years, 4 males; systemic sclerosis, *n* = 12, median (IQR), 62 (56–68) years, 5 males). We excluded patients with non-histologically confirmed diagnosis of lung cancer and with a follow-up inferior to 24 months for nintedanib treatment. We also excluded fibrotic HP patients without any histological confirmation diagnosis. The diagnosis of IPF was confirmed by multidisciplinary discussion. Adenocarcinoma was diagnosed histologically according to WHO 2015 criteria [21,22]. Twelve healthy controls (median age (IQR) 63 years (52–65); 5 males) were also enrolled.

Serial serum samples were collected from IPF patients before starting antifibrotic therapy (t0), and 12 (t1) and 24 months (t2) later. Lung function tests and high-resolution computed tomography (HRCT) of the chest were performed in all patients for diagnostic purposes. The former were repeated according to our center’s follow-up protocol and performed according to ATS/ERS standards [23] using a Jaeger body plethysmograph with corrections for temperature and barometric pressure. Forced expiratory volume in the first second (FEV1), FVC and diffusing capacity of the lung for carbon monoxide (DLCO) were recorded.

Demographic and clinical data, including comorbidities, family history, lung function parameters, and radiological features were collected from medical records and entered in an electronic database for statistical analysis. Healthy controls and patients gave their written informed consent to participation in the study, which was approved by our local ethics committee (C.E.A.V.S.E. Tuscany, Markerlung number 17431).

### 4.2. Krebs von den Lungen-6 Assay

Serum samples from all patients were assayed for KL-6 concentrations by KL-6 reagent assay (Fujirebio Europe, Gand, Belgium), as previously reported [6,24,25,26,27,28,29,30,31]. The principle of the assay is agglutination of sialylated carbohydrate antigen in samples with KL-6 mAb by antigen–antibody reaction. The change in absorbance was measured to determine KL-6 levels. Serum concentrations of KL-6 were expressed in U/mL.

### 4.3. Statistical Analysis

Data are presented as median and interquartile range (IQR). Non-parametric tests (Kruskal–Wallis test and Dunn’s multiple test) were used for comparisons among the four groups (IPF, IPF-LC, fHP and healthy controls). The Chi-squared test was used for categorical variables as appropriate. Serum concentrations of KL-6 were also compared between groups, assessing areas (AUC) under the receiver operating characteristic (ROC) curves. A *p*-value less than 0.05 was considered statistically significant. All statistical analysis and related figures were obtained using GraphPad Prism 9.0 software.

## 5. Conclusions

Lung cancer significantly reduces the survival of IPF patients. The association of lung cancer and IPF also has a negative effect on quality of life. The present study demonstrated higher KL-6 concentrations in IPF patients with lung cancer than in IPF patients without cancer. It confirmed KL-6 to be a reliable prognostic biomarker, predictive of IPF progression and indicative of response to nintedanib treatment. IPF patients treated with this drug for 24 months maintained stable FVC percentages and KL6 concentrations. KL-6 therefore also proved to be useful for monitoring antifibrotic therapy, besides enabling identification of the subgroup of IPF patients with lung cancer.

## Figures and Tables

**Table 1:** Main characteristics of our study population including age, gender, smoking habit, BMI, LFT parameters, and KL-6 serum concentrations. All data were expressed as median and interquartile range (IQR).

Parameters	All Patients (*n* = 142)	IPF (*n* = 10)	IPF-LC (*n* = 12)	fHP (*n* = 14)	PF-AD (*n* = 22)	HC (*n* = 12)
Age (median IQR)	69 (65–76)	77 (67–80)	72 (65–76)	67 (57–72)	63 (55–68)	63 (52–65)
Gender, M/F	86/56	8/2	8/4	8/6	9/13	5/7
Smoking habit (never/former)	73/69	4/6	5/7	6/8	8/14	8/4
BMI (kg/m^2^)	27 (25–28)	26 (23–29)	23 (21–26)	27 (24–30)	27 (25–29)	26 (24–27)
Pulmonary function parameters (median, IQR)						
FVC %	83 (75–91)	86 (72–101)	63 (50–76)	70 (58–84)	89 (79–102)	101 (88–107)
FEV1 %	83 (73–95)	87 (73–102)	63 (52–75)	66 (60–81)	85 (78–96)	98 (89–110)
DLCO %	55 (45–73)	49 (43–59)	25 (21–26)	55 (23–56)	56 (43–58)	90 (83–99)
KL-6 U/ml		1252 (977–1742)	1784 (1523–2242)	988 (883–1257)	1500 (846–2008)	211 (121–241) ^1^

Abbreviations: IPF, idiopathic pulmonary fibrosis; IPF-LC, idiopathic pulmonary fibrosis associated with lung cancer; fHP, fibrotic hypersensitivity pneumonitis; PF-AD, pulmonary fibrosis associated with autoimmune diseases; HC, healthy controls; KL-6, Krebs von den Lungen-6; BMI, body mass index; FVC, forced vital capacity; FEV1, forced expiratory volume in 1 s; DLCO, diffuse lung carbon monoxide. (1. HC vs. IPF-LC, fHP, IPF and PF-AD *p* < 0.0001) All statistical differences of KL-6 concentrations between disease groups were reported in the manuscript.

**Table 2:** Area under the receiver operating characteristics curve were performed between disease groups and healthy controls.

Serum KL-6 Concentrations	AUC	*p* Value	Cut-off Value	Sensitivity	Specificity
IPF-LC vs. HC	100	<0.0001	1137	100	92
IPF vs. HC	100	<0.0001	614	100	90
fHP vs. HC	100	<0.0001	649	100	93
PF-AD vs. HC	100	<0.0001	743	100	94

Abbreviations: IPF, idiopathic pulmonary fibrosis; IPF-LC, idiopathic pulmonary fibrosis associated with lung cancer; fHP, fibrotic hypersensitivity pneumonitis; PF-AD, pulmonary fibrosis associated with autoimmune diseases; HC, healthy controls; KL-6, Krebs von den Lungen-6.

## Data Availability

Data is contained within the article or supplementary material.

## Abbreviations

ILD	interstitial lung disease
IPF	idiopathic pulmonary fibrosis
IPF-LC	IPF associated with lung cancer
HRCT	High resolution computed tomography
KL-6	Krebs von den Lungen-6
fHP	fibrotic hypersensitivity pneumonitis
PF-AD	pulmonary fibrosis associated with autoimmune diseases
ROC	receiver operating characteristic
AUC	area under the curve
